# Chemotaxis Receptor Complexes: From Signaling to Assembly

**DOI:** 10.1371/journal.pcbi.0030150

**Published:** 2007-07-27

**Authors:** Robert G Endres, Joseph J Falke, Ned S Wingreen

**Affiliations:** 1 Department of Molecular Biology, Princeton University, Princeton, New Jersey, United States of America; 2 Department of Chemistry and Biochemistry, University of Colorado Boulder, Boulder, Colorado, United States of America; University of Auckland, New Zealand

## Abstract

Complexes of chemoreceptors in the bacterial cytoplasmic membrane allow for the sensing of ligands with remarkable sensitivity. Despite the excellent characterization of the chemotaxis signaling network, very little is known about what controls receptor complex size. Here we use in vitro signaling data to model the distribution of complex sizes. In particular, we model Tar receptors in membranes as an ensemble of different sized oligomer complexes, i.e., receptor dimers, dimers of dimers, and trimers of dimers, where the relative free energies, including receptor modification, ligand binding, and interaction with the kinase CheA determine the size distribution. Our model compares favorably with a variety of signaling data, including dose-response curves of receptor activity and the dependence of activity on receptor density in the membrane. We propose that the kinetics of complex assembly can be measured in vitro from the temporal response to a perturbation of the complex free energies, e.g., by addition of ligand.

## Author Summary

Chemotaxis allows bacteria to sense and swim toward nutrients and away from toxins. The remarkable sensing properties of the chemotaxis network, such as high sensitivity to small changes in the chemical environment, are thought to originate from receptor complexes in the membrane, which act as antennas to magnify weak signals. To adapt to persistent stimulation, receptors are covalently modified. While the individual protein components of the chemotaxis network are well characterized, making the system well suited for quantitative and computational analysis, direct experimental visualization of receptors and receptor complexes is difficult within the current limits of fluorescence and electron microscopy. To address questions such as how large are complexes and why do they assemble, we analyze in vitro signaling data using a previously developed model of signaling by receptor complexes. Based on the data, we propose a statistical physics model for the distribution of complex sizes in the membrane. Within this model, complex size depends on the receptor free energy with contributions from receptor modification level, ligand binding, receptor–receptor coupling, and binding to accessory proteins. Our model results compare favorably with a variety of different signaling data, and suggest new experiments to measure the kinetics of assembly of receptor complexes.

## Introduction

The chemotaxis network allows bacteria to sense and swim toward attractants (nutrients such as amino acids and sugars) and away from repellents. For this purpose, cells are equipped with ∼10,000 chemoreceptors, forming large arrays at one or both cell poles. The chemotaxis network has remarkable properties, including signal integration by multiple types of chemoreceptors [1], precise adaptation to persistent stimulation [2,3], and high sensitivity to changes in ligand concentration [1] over several orders of magnitude of background concentrations. These signaling properties are thought to originate from strongly coupled receptor complexes [4,5]. Specifically, in vivo fluorescence resonance energy transfer (FRET) measurements of receptor sensitivity [1] and Hill coefficients [6] indicate coupled complexes of up to 10–20 receptor homodimers [6–10]. Despite the importance of complex size to signaling, little is known about what controls receptor complex size (for recent reviews see [11,12]). In vivo observation of complex size and dynamics, e.g., by fluorescence recovery after photobleaching (FRAP), is currently not practical because of limited spatial resolution. However, the close relation between complex size and the sensitivity and cooperativity of signaling means that receptor activity can be used to probe complex size [8]. To demonstrate the potential of this approach, we analyze in vitro receptor-activity data [13–15] and present a simple biophysical model for the energetics of complex assembly.

Here we mainly focus on data from Bornhorst and Falke [13], whose in vitro receptor-activity assay employed a chemotaxis null strain of Escherichia coli overexpressing one of the five receptor types, the high-abundance receptor Tar. The Tar receptor specifically binds aspartate and its nonmetabolizable analogue methyl-aspartate (MeAsp). The cytoplasmic membranes were isolated, and incubated with purified CheW, CheA, and CheY proteins. In vivo, CheW enhances complex formation and mediates binding to the kinase, CheA. Active CheA autophosphorylates using ATP and transfers the phosphate to the response regulator, CheY. Phosphorylated CheY diffuses to the flagellar motor and induces clockwise rotation and cell tumbling. In vitro, CheA kinase activity was measured by assaying the rate of phosphorylation of CheY using radiolabeled ATP. CheA activity is inhibited by an increase of attractant concentration. For the assay, receptors were genetically engineered to have either a glutamate (E) or a glutamine (Q) at each of four specific modification sites in the cytoplasmic domain. In vivo, these four modification sites are used for adaptation, with the enzyme CheR methylating glutamates to increase the kinase activity, and the enzyme CheB demethylating methylated glutamates to decrease the kinase activity. In chemotaxis, a Q is functionally similar to a methylated E. For instance, Tar{QQQQ} is highly active at zero attractant concentration, while Tar{EEEE} is generally inactive.

**Figure 1:**
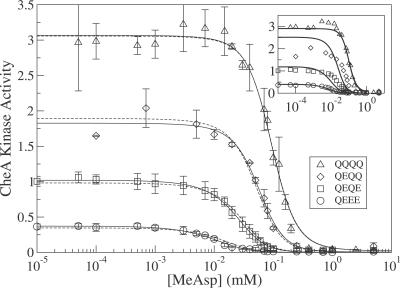
In Vitro Dose-Response Curves and Fits from Models Activity of CheA complexed to Tar receptors of defined modification state (QQQQ, QEQQ, QEQE, QEEE) for different MeAsp attractant concentrations. Data (mean and standard deviation of three measurements) are taken from in vitro activity assays of Bornhorst and Falke [13]. All measurements used the same total amount of receptor. Least-squares fits: solid curves, simple model of signaling by trimers of dimers, where the amplitude of each curve (e.g., QQQQ) is an independent fitting parameter; dashed curves, equilibrium-assembly model of an ensemble of single dimers, dimers of dimers, trimers of dimers, and CheA-bound trimers of dimers, where only CheA-bound trimers of dimers can signal. Inset: all receptors assumed to form trimers of dimers. The MeAsp dissociation constants *K_D_* ^off^ = 0.02 mM and *K_D_* ^on^ = 0.5 mM are taken from [8].

Figure 1 shows experimental in vitro dose-response curves from Bornhorst and Falke [13], i.e., CheA activity versus stimulation by different amounts of attractant, for Tar receptors in defined modification states. Hill coefficients are smaller (and sensitivities are lower) than typical for in vivo studies of cells overexpressing Tars [6,8], indicating smaller in vitro clusters. The in vitro Hill coefficients (*n_H_* ≈ 2–3) are in line with expectations from partial crystal structures [16] and cross-linking experiments [17,18] indicating that receptors oligomerize into mixed trimers of homodimers as the smallest unit of complexes. In vivo, larger complexes possibly form with a hexagonal lattice structure [19,20]. Modeling in vitro data using receptor complexes of a single fixed size (e.g., trimers of dimers) does not describe the data well (inset Figure 1). Here we examine a model in which the receptor modification state determines the amount of trimers of dimers, yielding a significantly better fit to the data (solid lines in Figure 1) and suggesting that receptor modification may vary complex size, possibly along with other parameters [21].

In this paper, we analyze in detail the in vitro activity data from Bornhorst and Falke [13], Shrout et al. [14], and Lai et al. [15]. We model homodimers of Tar receptors in membranes as an ensemble of different species, including single dimers, dimers of dimers, trimers of dimers, and the signaling complex formed by the kinase CheA bound to trimers of dimers, in line with recent experiments [22]. The relative free energies of these species determine their equilibrium distribution, accounting for the different amounts of actively signaling trimers of dimers indicated by the data. We further propose that the kinetics of receptor-cluster assembly can be measured experimentally by perturbing the receptor free energies, e.g., through addition of ligand.

## Results

The experimental dose-response curves in Figure 1 for Tar receptors in different modification states were obtained from in vitro reaction mixtures which always contained the same total amounts of receptor, adapter protein CheW, kinase CheA, and response regulator CheY [13]. Addition of MeAsp inhibits the kinase activity, while the number of Qs per receptor increases the kinase activity. Previously, similar dose-response curves from living cells, obtained by in vivo fluorescence resonance energy transfer (FRET), were successfully modeled using the Monod–Wyman–Changeux (MWC) model [23] of strongly coupled two-state receptors [24], and revealed complex sizes of order *N* = 10 receptors [6–10]. Here we employ the same MWC model to estimate the size of receptor complexes in the in vitro assays of Bornhorst and Falke.

In the MWC model, the receptor complex activity is simply the probability for the complex to be on, which is fully determined by the free-energy difference between on and off states of the complex (Equation 1). For a homogenous complex of Tar receptors, this free-energy difference is the product of the number of receptors, *N*, in the complex and the free-energy difference between on and off states of a single Tar receptor. The free-energy difference of a single receptor has two contributions. One contribution, Δɛ(*m*), depends on receptor modification level, *m*, and ranges from positive for fully demethylated (*m* = 0) receptors to negative for fully methylated (*m* = 8) receptors. The other contribution arises from attractant binding and depends on the ligand dissociation constants 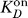 and 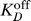 of the on and off states, respectively. If the activity is low in the absence of ligand (e.g., for demethylated receptors), the inhibition constant (ligand concentration at half maximal activity) is *K_i_* ≈ 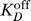 /*N* and the Hill coefficient is *n_H_* ≈ 1. In contrast, if the activity is high in the absence of ligand (e.g., for highly methylated receptors), the inhibition constant is 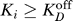 and the Hill coefficient is *n_H_* ≈ *N*, where *N* is the number of receptors in the complex ([8], see Methods). Inspection of the experimental dose-response curves in Figure 1 shows that the inhibition constant of the low-activity QEEE curve is about K*_i_* ≈ 0.01 mM MeAsp and that Hill coefficients of the other curves are *n_H_* ≈ 2–3. Hence, based on the MWC model and the previously determined value 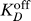 = 0.02 mM for Tar receptors binding MeAsp [8], the signaling complexes responsible for the data in Figure 1 are likely to be trimers of dimers.

Indeed, the MWC model using *N* = 3 for trimers of dimers and a different Δ∈(*m*) for each receptor modification state *m* (Equations 1 and 2) fits the shapes of the in vitro curves well, while allowing each curve to have a free amplitude *α_m_* (solid curves in Figure 1). However, in the MWC model, Δ∈(*m*) is also supposed to determine the relative amplitudes of the curves. Although amplitudes still depend systematically on the number of Qs (*m*), the relative amplitudes from the MWC model are substantially different and do not describe the data well (inset in Figure 1). Hence, each dose-response curve is well described by the MWC model for trimers of dimers, but the MWC model does not describe the relative amplitudes correctly. (Use of a two-state model without cooperativity [21] or use of an alternative MWC model with a methylation-dependent 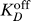 to fit experimental amplitudes both produce lower than observed Hill coefficients.) The discrepancy in amplitudes raises the following question—given that all experiments use the same total amount of receptor, why should the amplitudes systematically differ from the MWC model predictions for different receptor-modification states?

According to recent in vitro experiments, only receptors in trimers of dimers can signal [22]. Therefore, the presence of some receptors as (inactive) single dimers and dimers of dimers could account naturally for the different amplitudes observed in Figure 1. We therefore suggest that in the in vitro assays not all receptors form trimers of dimers, some also partition into single dimers and dimers of dimers, with the fraction in trimers of dimers depending on the receptor-modification state. In fact, such a partition is required by thermodynamic equilibrium, with entropy favoring single dimers and dimers of dimers over trimers of dimers. In the following, we formulate an equilibrium model to predict the amounts and activities of trimers of dimers as a function of receptor-modification state. For this purpose, we include CheA binding to trimers of dimers only, leading to an equilibrium between free trimers of dimers, without signaling capability, and CheA-bound trimers of dimers, the signaling complex. (For simplicity, we assume that CheW is present at saturation.)

**Figure 2:**
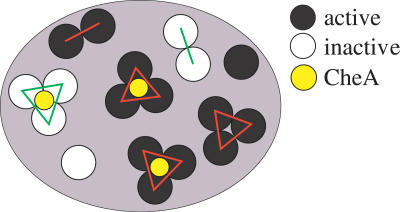
Schematic of Membrane-Bound Chemoreceptor Complexes Membrane contains equilibrated mixture of active (black) and inactive (white) single dimers, dimers of dimers, trimers of dimers, and CheA-bound trimers of dimers, where CheA is shown in yellow. Only CheA-bound trimers of dimers can signal. Red and green lines indicate interactions among active and inactive receptors, respectively.

In our model for Tar receptors in membranes, we consider single dimers, dimers of dimers, trimers of dimers, and CheA-bound trimers of dimers. These different species can either be active (on) or inactive (off) as illustrated in Figure 2, but only active CheA-bound trimers of dimers can signal. The relative free energies of the various species determine their equilibrium distribution. To compare the free energies of the different species, we introduce homodimer–homodimer coupling energies, which can be different between active homodimers (*J*_on_) and between inactive homodimers (*J*_off_). We also include a chemical potential, *μ*, to adjust the receptor density. The resulting free-energy expressions are given in Equations 3–10. To facilitate calculations, we treat the membrane as a lattice where each site can be either empty, or occupied by a single dimers, a dimer of dimers, a trimer of dimers, or a CheA-bound trimer of dimers, yielding the partition function in Equation 11. To model the in vitro experiments, in which the same total amount of receptor was used for each assay, we multiply the probability that a given CheA-bound trimer of dimers is active by the fraction of receptors in CheA-bound trimer of dimers (cf. Equation 14 in Methods).

This equilibrium-assembly model (dashed lines in Figure 1) describes the data as well as the ad hoc model with free amplitudes (solid lines in Figure 1). Specifically, the equilibrium-assembly model accounts for the systematic dependence of the dose-response curve amplitudes on receptor modification state. Since for each curve we assume a fixed fraction of CheA-bound trimers of dimers, set by the incubation conditions, the shape of each curve is still determined by the MWC model with *N* = 3 (Equation 13 in Methods). While the equilibrium-assembly model requires seven parameters, 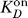 , 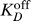 , *J*_on_, *J*_off_, ∈*_A_*, *μ*, and *α*, plus an offset energy, Δ∈, for each receptor-modification state, some of these parameters are nearly redundant. For example, Δ∈ and *J*_on_ − *J*_off_ play nearly equivalent roles, as do *μ* and (*J*_on_ + *J*_off_)/2, differing only in their effects on the ratio of dimers of dimers and trimers of dimers. Therefore, our parameter choices represent only one consistent set of values.

**Figure 3:**
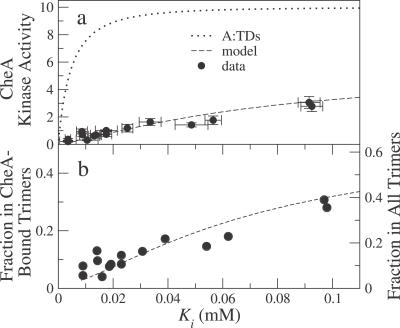
Kinase Active Receptors Are in CheA-Bound Trimers of Dimers (A) CheA kinase activity at zero MeAsp concentration versus *K_i_*, defined as the MeAsp concentration required to reduce activity by one-half, for Tar receptors in different modification states. Data (mean and standard deviation of three measurements) are taken from in vitro activity assays and Hill fits of Bornhorst and Falke [13]. All measurements used the same total amount of receptor. Dotted curve, all receptors assumed to form CheA-bound trimers of dimers (A:TDs), with maximal activity *α* = 10. Dashed curve, equilibrium-assembly model with same *α* (cf. Figure 1, dashed curves). (B) Left axis, fraction of receptors in A:TDs, i.e., ratio of data or dashed curve to the dotted curve in (A). Right axis, fraction of receptors in all trimers (TDs and A:TDs), equal to the fraction of receptors in A:TDs multiplied by 1 + exp(∈*_A_*), where ∈*_A_* is the binding energy of CheA (see Methods). The MeAsp dissociation constants *K_D_* ^off^ = 0.02 mM and *K_D_* ^on^ = 0.5 mM are taken from [8].

In their data, Bornhorst and Falke [13] observed a strong correlation between the activity in the absence of MeAsp and the inhibition constant *K_i_*. Figure 3A shows this correlated data for all possible modification states except EEEE, for which the measured activity was zero. The observed functional relation between activity and *K_i_* supports our suggestion that not all receptors form CheA-bound trimer of dimers. To illustrate, in Figure 3A we have plotted, as a dotted curve, the expected relation between activity and *K_i_* if all receptors did form CheA-bound trimers of dimers. The curve has a noticeably different shape from the experimental data. In contrast, the equilibrium-assembly model, with the same parameters as in Figure 1, is able to capture the observed relation between activity and *K_i_* (dashed curve). In either case, the one-to-one relation between activity and *K_i_* follows because both quantities depend uniquely on the receptor offset energy Δ∈. For ease of comparison, we used the same amplitude parameter *α* = 10 for both curves in Figure 3A. This means that the ratio of the two curves gives the fraction of receptors in CheA-bound trimers of dimers in the equilibrium-assembly model, because only those receptors in CheA-bound trimer of dimers contribute to the activity. The actual fraction of receptors in CheA-bound trimers of dimers (and in all trimers) is shown in Figure 3B, both for the equilibrium-assembly model and, by inference, for the in vitro data. Why does the fraction of receptors in CheA-bound trimers of dimers increase with *K_i_*? Within the model, the inhibition constant *K_i_* increases as the offset energy Δ∈ decreases; this behavior follows because decreasing Δ∈ favors the active state of receptors, and therefore more attractant is required to inactivate them. The same shift of receptors toward higher activity causes the fraction of receptors in CheA-bound trimers of dimers to increase, both because *J*_on_ < *J*_off_ implies a stronger tendency of active receptors to form trimers of dimers, and simply because increasing the total concentration of active receptors increases their equilibrium partition into trimers of dimers.

**Figure 4:**
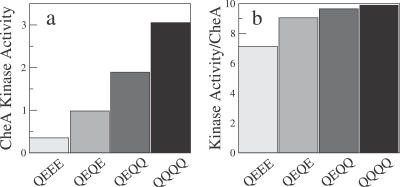
Kinase Activity per Receptor-Bound CheA (A) CheA kinase activity at zero attractant concentration for four different receptor modification states within the equilibrium-assembly model (Figure 1, dashed curves). (B) Kinase activity per receptor-bound CheA, i.e., activity from (A) divided by fraction of receptors in CheA-bound trimers of dimers (cf. Figure 3B).

Our suggestion that not all receptors form trimers of dimers or CheA-bound trimers of dimers is given further experimental support by Shrout et al. [14] and Lai et al. [15] who used a receptor-activity assay similar to that of Bornhorst and Falke but with E. coli Tar receptors. Shrout et al. measured the kinase activity for different modification states of cytoplasmic Tar-receptor fragments at zero attractant concentration. While the measured activities depended strongly on modification state, the same activities normalized by the amount of bound CheA were almost independent of modification state. We find the same behavior in our equilibrium-assembly model. Figure 4 shows the calculated activity and activity per CheA (activity divided by the fraction of receptors in CheA-bound trimers of dimers) for four different receptor-modification states (cf. Figure 1). We observe qualitative agreement with the data in Figure 2A of Shrout et al. [14], although their receptor fragments tend to be more active than complete receptors [25]. In the equilibrium-assembly model, if the CheA-bound trimers of dimers were always fully active (on), the normalized activities would be completely independent of the modification state. However, for receptors with few Qs, the CheA-bound trimers of dimers are not fully active even at zero attractant concentration, resulting in the weak modification-level dependence of the normalized activity seen in Figure 4B.

**Figure 5:**
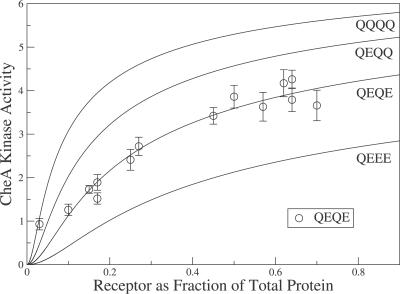
Kinase Activity versus Receptor Fraction of Total Membrane Protein The data for Tar{QEQE} receptors (mean and standard deviation of three measurements) are taken from in vitro activity assays of Lai et al. [15]. All measurements used the same amount of receptor. Solid curves for receptors in different modification states were calculated with parameters obtained from the equilibrium-assembly model (Figure 1, dashed curves). Calculated activities are scaled by 0.9 to convert to activity of Lai et al. [15], and receptor density is scaled by 5.3 to convert to receptor as a fraction of total protein.

If an equilibrium exists among single dimers, dimers of dimers, trimers of dimers, and CheA-bound trimers of dimers, one would expect changes in the receptor density to affect the distribution of different sized receptor clusters. Consistent with this expectation, Lai et al. [15] reported the activity per Tar{QEQE} receptor, in the absence of attractant, as a function of the receptor fraction of total membrane protein. As shown in Figure 5, they observed an increase in and saturation of the activity per receptor with increasing receptor fraction. We interpret their data to mean that at low receptor fractions (densities), it is thermodynamically unfavorable for receptors to come together and form trimers of dimers (or even dimers of dimers), and consequently single dimers, which lack signaling capability, predominate. This density-dependent activity per receptor is captured by our equilibrium-assembly model, as shown in Figure 5 (solid lines), using the same parameters as in Figure 1. The calculated activity is scaled by an overall factor to convert to the activity scale of Lai et al. [15], and the calculated receptor density (Equation 15) is also rescaled. Within the equilibrium-assembly model, the kinase activity per receptor increases with receptor density entirely because of the increasing fraction of receptors in CheA-bound trimers of dimers expected from thermodynamics.

**Figure 6:**
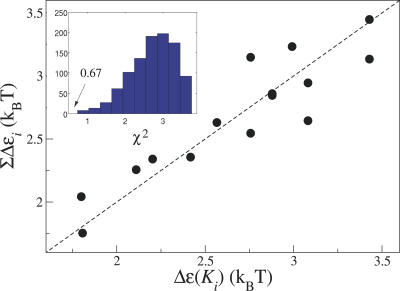
Additivity of Offset Energies from Four Modification Sites Test of additivity of the contributions of the four modification sites to the total offset energy Δ∈, based on the in vitro data for 15 receptor modification states (all possible combinations of Es and Qs, except EEEE) from Table I of [13]. Horizontal axis, Δ∈ obtained from experimental *K_i_* values (see Methods); vertical axis, Δ∈ determined from the sum of the offset energies from the four individual modification sites Δ∈*_i_* treated as χ^2^ fitting parameters (see Methods). Inset, histogram of χ^2^ values after minimization for 1,000 randomized permutations of the data; χ^2^ = 0.67 of the unrandomized data is the lowest χ^2^ (see Methods).

The large amount of in vitro data from Bornhorst and Falke [13] can be used to test an additional hypothesis. Specifically, do the offset energies from each of the four modification sites Δ∈*_i_*_=1,2,3,4_ contribute additively to give the total offset energy Δ∈? The total offset energy Δ∈ for each of the 15 modification states can be obtained from the inhibition constants *K_i_* [13] based on our model that only CheA-bound trimers of dimers can signal (see Methods). This value can be compared with the additive model, where the Δ∈*_i_* are treated as fitting parameters. Figure 6 shows that the additive model for the total offset energy is indeed a reasonably good approximation. Interestingly, modification sites 1 to 3 make a similar contribution (approximately −0.5 to −0.6 *k_B_T* ) while site 4 makes a smaller contribution (approximately −0.3 *k_B_T*) to the offset energy (see Methods). This may have to do with the fact that, relative to the CheA binding site, modification sites 1 to 3 are nearby on the N-terminal side of the receptor and modification site 4 is on the C-terminal side of the receptor.

## Discussion

The chemotaxis network of E. coli exhibits remarkable sensing and signaling properties that rely on receptor complexes. Despite recent high resolution electron microscopy [19,20], fluorescence images [26–28], and in vivo fluorescence recovery after photobleaching (FRAP) measurements of protein dynamics (V. Sourjik, personal correspondence), very little is known about what determines receptor-complex size [11,12]. Interestingly, because complex size and signaling sensitivity or cooperativity are closely related [8], receptor kinase activity can be used to probe complex size. Starting from in vitro dose-response data of the activity of Tar receptors in native membranes [13–15], we presented a simple biophysical model for the energetics of complex assembly that can account for these and other data. An essential feature of the model is that not all receptors form signaling complexes, i.e., kinase CheA-bound trimers of dimers. Our model for receptor complexes is based on an MWC model, with constants 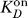 and 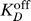 , in which receptor modification state affects complex size only through the offset energy Δ∈ (which depends additively on contributions from the four modification sites). At this stage, we cannot rule out alternative models, e.g., in which modification state affects other parameters as well [21].

In our model, Tar receptors form an ensemble of different species, including single dimers, dimers of dimers, trimers of dimers, and CheA-bound trimers of dimers, as illustrated in Figure 2. The different species can either be active (on) or inactive (off), but only active CheA-bound trimers of dimers can phosphorylate CheY. This is in line with recent in vitro experiments where trimers of dimers were found to signal, but single dimers and dimers of dimers did not signal [22]. The relative free energies of the various species determine their equilibrium distribution, leading naturally to the observed variation in the signaling activity of receptors in different modification states (cf. Figures 1, 3, 4, 5). We find that the fraction of receptors in trimers of dimers and CheA-bound trimers of dimers increases with the number of Qs at the modification sites (or with *K_i_*, see Figure 3B). Within this picture, the “superactivity” of certain mutant receptors can be attributed to more efficient complex formation rather than enhanced CheA binding or kinase velocity [25].

**Figure 7:**
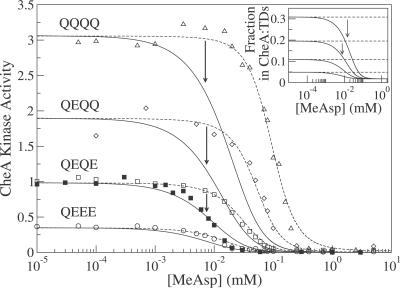
From Signaling to Assembly Kinetics Difference between the equilibrium partition of receptors incubated without MeAsp (dashed curves) or with MeAsp (solid curves), using the equilibrium-assembly model. Dashed curves and data (open symbols) are from Figure 1. Experimentally, the kinetics of complex assembly can be obtained from the ratio of relaxation from the dashed curves to the solid curves following addition of MeAsp (arrows). Also shown is data for Tar{QEQE} receptors from in vitro activity assays of Lai et al. [15] (solid squares), based on addition of MeAsp during incubation, but using different protein concentrations compared with Bornhorst and Falke [13]. Inset. fraction of receptors in CheA-bound trimers of dimers (CheA:TDs).

Our free-energy model assumes that complex assembly/disassembly is slow compared with changes in signaling. For instance, if attractant is added together with ATP to initiate the activity measurement, the ensemble of clusters is assumed to stay frozen, i.e., the ratio of {single dimers}:{dimers of dimers}:{trimers of dimers}:{CheA-bound trimers of dimers} is assumed to be unaffected by the addition of attractant, even though the kinase activity is immediately affected. This separation of time scales is reflected in Equation 14, where the fraction of receptors in CheA-bound trimers of dimers (first factor) is evaluated at the incubation attractant concentration ([L_0_] = 0), while the activity (second factor) is evaluated in the presence of the added attractant ([L]). To model the case where attractant is added during incubation, one only needs to set [L_o_] = [L]. In this case, shown by solid curves in Figure 7, inhibition occurs at lower attractant concentrations, in agreement with the data of Lai et al. [15] for Tar{QEQE} incubated in the presence of MeAsp (solid symbols). In the model, the inhibition at lower attractant concentrations can be traced to the loss of trimers of dimers in favor of single dimers and dimers of dimers in the new equilibrium produced by incubation with attractant (see inset Figure 7). Incubation with attractant is exactly the opposite of adding Qs in terms of receptor free energies, and therefore favors smaller rather than larger complex sizes.

The dose-response curves in Figure 7 for incubation without attractant (dashed curves) and with attractant (solid curves) are easily distinguishable, which suggests a way to measure the kinetics of complex assembly. During the period after the addition of attractant, as the clusters re-equilibrate, the dashed curves must evolve toward the solid curves. The rate of evolution can be quantified by measuring the kinase activity at specific times following the addition of attractant. In this way, information can be obtained about the kinetics of assembly and disassembly of receptor complexes. Our equilibrium-assembly model, augmented by kinetic rate constants, provides an appropriate theoretical framework for planning and interpreting kinetic experiments of this type.

There are previously published models for chemoreceptor complex assembly. These models, however, do not consider the effects of ligand binding, and hence cannot address dose-response data. Furthermore, Lai et al. [15] assume all receptors form trimers of dimers, hence their model cannot explain the activity versus receptor density data in Figure 5. Shrout et al. [14] assume that CheA binding directly depends on the receptor modification state. While this assumption can explain the increase of activity with modification level, it violates the conventional view of precise adaptation based on the two-state receptor model, where receptors are either on (active) or off (inactive). Precise adaptation occurs because receptor modification responds exclusively to receptor activity so as to exactly balance the effects of ligand binding. If CheA binding depended directly on receptor modification level, this would increase kinase activity at higher attractant concentrations and, hence, interfere with precise adaptation. In contrast, in our model, CheA binds to trimers of dimers irrespective of modification level or activity. The recent model by Asinas and Weis [25] considers the competitive assembly of wild-type and activity-mutant receptors. The authors come to a similar conclusion to ours, i.e., that receptor activity determines cluster assembly and, consequently, CheA recruitment and activity (see also Li and Weis [29]).

An approach similar to ours may allow measurement of the kinetics of receptor complexes in living cells. Complex sizes of 10–20 receptors or more have been inferred from in vivo dose-response curves [6–10] and, in E. coli cells lacking an adaptation system, polar clustering appears to depend on receptor-modification level ([28,30,31]; V. Sourjik, personal correspondence). This suggests that dose-response curves can be used to measure the real-time evolution of in vivo cluster sizes in response to perturbations of receptor free energy, e.g., addition of attractant or repellent. It is not clear why in vivo complexes are significantly larger than the trimers of dimers seen in vitro and why receptors localize predominately at the cell poles. It is known that receptors are inserted into the membrane by the Sec translocon machinery [32] in large cell-spanning spirals [33]. Once inserted into the membrane, receptors may localize at the cell poles due to the higher membrane curvature [34] and/or different lipid composition [35–37] at the poles. A means to probe receptor-assembly kinetics may help reveal what determines complex size in vivo.

Compared with previous modeling of in vivo data [8–10], the offset energies, Δ∈, obtained from in vitro data are much larger. This can be traced to the fact that we explicitly include homodimer–homodimer interactions, which lead to an effective offset energy for each receptor in a trimer of dimer of Δ∈ + *J*_on_ − *J*_off_, close to estimated in vivo values. However, in a large in vivo complex, if each receptor participates in six homodimer–homodimer interactions, as on a hexagonal lattice, the effective offset energy per receptor would be Δ∈ + 3(*J*_on_ − *J*_off_), which is much more negative than the estimated in vivo values. One possible resolution might be that, in an in vivo cluster, homodimers in different trimers of dimers are coupled together more weakly than homodimers within a trimer of dimers. However, the coupling between trimers of dimers must still be strong enough to cause clusters of 10–20 receptors to switch on and off together. An important open question is what mediates the interactions among receptor homodimers in trimers of dimers, or between trimers of dimers? One way to address this question may be to measure in vitro or in vivo dose-response curves of mutant receptors specifically engineered to interrupt or strengthen homodimer–homodimer interfaces. Possible insight can be gained from the observation of large in vitro Tsr clusters [29], pointing toward a difference between Tsr:Tsr and Tar:Tar interfaces [15].

We expect that a better understanding of the assembly of E. coli chemoreceptor complexes may provide insights into the oligomerization of other membrane proteins, including bacterial outer membrane proteins such as porins (e.g., LamB). For other membrane-bound receptors that form complexes, including ryanodine receptors [38,39] and rhodopsin [40], we hope that analysis of complex size and assembly kinetics based on dose-response curves may also prove feasible.

## Methods

### Review of in vitro activity assay.

We mainly model the data of Bornhorst and Falke [13], who used an in vitro activity assay to study chemotaxis signaling. Briefly, Tar receptors of Salmonella typhimurium were engineered to be in a particular modification state, e.g., QQQQ, QEQQ, QEQE, or QEEE, where Q is approximately equivalent to a methylated E. Using a chemotaxis null strain of E. coli, the Tar receptor was overexpressed from a plasmid. Cytoplasmic membranes were isolated in which Tar receptors constituted approximately 5%–10% of total membrane protein. Reaction mixtures of the same total amount of Tar and purified CheA, CheW, and CheY were prepared and incubated for 45 min to allow for complex formation in native membranes. Signaling was initiated by adding radiolabeled ATP. The activity of CheA was measured by assaying the rate of phosphorylation of CheY and normalized to QEQE (wild-type). Quantified attractant (MeAsp) was added with the ATP.

### Monod–Wyman–Changeux model.

In the MWC model [6,8,23], two-state receptors (homodimers) [24,41] form complexes with all receptors in a complex either on or off together. At equilibrium, the probability that an MWC cluster of *N* Tar receptors will be active is

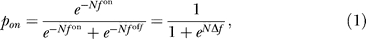
where *N f* ^on^ (*N f* ^off^) and *f* ^on^ (*f* ^off^) are the free energies of the complex as a whole and an individual receptor to be on (off), respectively. The individual receptor free-energy difference is given by

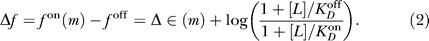



Here, [L] is the ligand (MeAsp) concentration, *m* is the number of Qs per receptor (*m* = 0,…,8), and 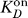 and 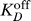 are the ligand dissociation constants in the on and off states, assumed to be independent of *m*. All energies are expressed in units of the thermal energy *k_B_T*.

In our model, Qs or methylated Es favor the on state of a receptor by lowering Δ∈ (*m*), while attractant binding favors the off state, i.e., 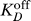 < 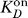 . Importantly, the model exhibits two regimes [8]. In regime I, where Δ∈ > 0 (e.g., for Tar{EEEE}), receptors have a low activity and an inhibition constant (ligand concentration at half maximal activity), *K_i_* ≈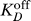 /*N*, indicating an *N* times higher sensitivity than for a single receptor. In regime II, where Δ*∈* < 0 (e.g., for Tar{QQQQ}), receptors are highly active, and turn off at large attractant concentration *K_i_* ≈ 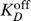 exp(|Δ*∈*|) with high cooperativity, i.e., a Hill coefficient *n_H_ ≈ N*. The possible MWC complexes considered here are the single receptor dimer, the dimer of dimers, and the trimer of dimers, corresponding to complex sizes *N* = 1, 2, and 3, respectively.

### Equilibrium-assembly model.

We use statistical mechanics to predict the partitioning of receptors into active and inactive single dimers, dimers of dimers, trimers of dimers, and CheA-bound trimers of dimers, as illustrated in Figure 2. Since CheA-bound trimers of dimers are the signaling complex, only trimers of dimers can signal, not single dimers and dimers of dimers, in line with recent experiments [22]. To compare the energies of the different-sized complexes, we generalized the MWC model to include homodimer–homodimer interactions. The interaction energy between active homodimers (*J*_on_) and the interaction energy between inactive homodimers (*J*_off_) can be different. These homodimer–homodimer interactions may originate from interactions of the periplasmic or cytoplasmic domains of the receptors, possibly mediated by the adapter protein CheW. Specifically, single dimers, dimers of dimers, and trimers of dimers (as well as CheA-bound trimers of dimers) have zero, one, and three homodimer–homodimer interactions, respectively. We also introduce a receptor chemical potential, *μ*, which determines the receptor density, *ρ,* in the membrane, and a free energy, *∈_A_*, for the binding of the kinase CheA to trimers of dimers (assuming for simplicity an equilibrium between bound CheAs and free CheAs at some invariant concentration). The resulting complex free energies for a single dimer (SD), a dimer of dimers (DD), a trimer of dimers (TD), and a CheA-bound trimer of dimers (A:TD) are given by

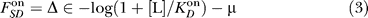


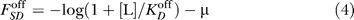


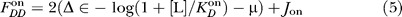


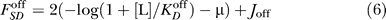


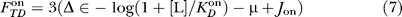


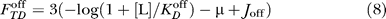


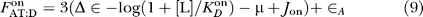


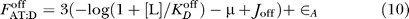



To regularize our calculations, we treat the membrane as a lattice where each lattice site can be either empty, or occupied by a single dimer, a dimer of dimers, a trimer of dimers, or a CheA-bound trimer of dimers, determined by their relative free energies. The equilibrium partition function of a single lattice site is given by

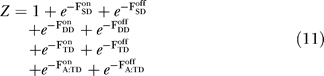



The probability that a site is occupied by species s (= SD, DD, TD, or A:TD) is given by

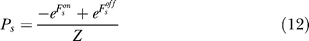



The probability that a particular CheA-bound trimer of dimers is active is given by the MWC model (cf. Equation 1), now also depending on *J*_on_ − *J*_off_,

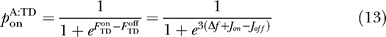



To compare with experiments on a per receptor basis, the probability that each CheA-bound trimer of dimers is active needs to be multiplied by the fraction of receptors in CheA-bound trimers of dimers, i.e.,

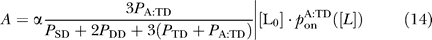
where *α* is an overall amplitude parameter, and can be interpreted as the maximal possible activity, which would be achieved if all receptors were in active CheA-bound trimers of dimers. The ligand concentrations [L_0_] and [L] indicate that the ensemble of species can equilibrate at one ligand concentration, e.g., [L_0_] = 0, while signaling can be measured at another concentration, [L].

Assuming a constant density, *ρ*, of receptors in the membrane, we find the chemical potential, *μ*, that yields this density. The definition of the density,

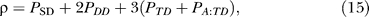
can be solved for *μ* by solving the cubic equation

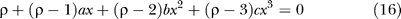
for *x* = *e^μ^* and choosing the largest real root. The coefficients are given by

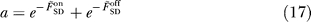


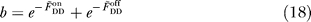


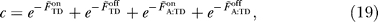
where 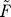 indicates that the chemical potential is removed from the free energy *F*, i.e., 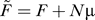 . The resulting chemical potential *μ* can be used in Equation 14 to calculated the activity.

Given 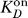 = 0.5 mM and 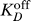 = 0.02 mM [8], Equation 14 for the activity depends on amplitude parameter *α* and five additional parameters: the second factor (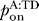 ) depends on Δ∈(*m*) and *J*_on_ − *J*_off_, and the first factor (fraction of receptors in CheA-bound trimers of dimers) depends additionally on *ρ*(*μ*), *J*_on_ or *J*_off_, and ∈*_A_*. We chose *α* = 10, somewhat above the activity 5.5 observed for superactive receptor mutants, to constrain the other energy parameters to be of reasonable size, i.e., on the order of *k_B_T*. Fitting the four dose-response curves in Figure 1 provides Δ∈(QEEE) = 3.1, Δ∈(QEQE) = 2.6, Δ∈ (QEQQ) = 2.2, Δ∈(QQQQ) = 1.8, ∈*_A_* = −1.32, *J*_on_ = 0.01, and *J*_on_ − *J*_off_ = −3.39 in units of *k_B_T*, and *ρ* = 0.045 receptors per site. For comparison, constraining the values to *J*_on_ = *J*_off_ leads to a fit as poor as that in the inset to Figure 1.

### Additivity of offset energies.

We test whether modifications of the four receptor sites contribute additively to the total offset energy Δ∈. From the measured inhibition constants, *K_i,_* of 15 different receptor modification states (QEEE, etc., except EEEE) [13], the total Δ∈ can be calculated from Equation 13, assuming only CheA-bound trimers of dimers can signal

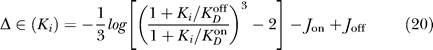
with *J*_on_ − *J*_off_ = −3.39 from the previous paragraph. These values for the total offset energies can be compared with the corresponding values within an additive model

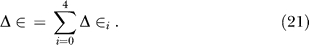
where Δ∈*_i = 1,…,4_* is the contribution to the total offset energy from the presence of a Q at site *i*. The values Δ∈*_i = 0,…,4_* are treated as fitting parameters obtained from minimizing

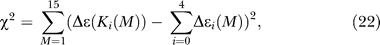
With *M* indexing the 15 modification states. Δ∈_0_ allows a fully unmodified receptor EEEE to have a nonzero offset energy. The best fit parameters are Δ∈_0_ = 3.738, Δ∈_1_ = −0.603, Δ∈_2_ = −0.504, Δ∈_3_ = −0.589, Δ*ɛ*∈ = −0.289. The resulting total offset energies, Δ∈(QEEE) = 3.135, Δ∈(QEQE) = 2.546, Δ∈(QEQQ) = 2.257, and Δ∈ (QQQQ) = 1.753, compare well with the values from the previous paragraph. All energies are given in units of the thermal energy, *k_B_T*.

To further test whether the additivity assumption is valid, we permutated the data, i.e., randomly reassigned all 15 *K_i_* values to the 15 different receptor modification states, and calculated *χ* ^2^ values after minimization. One thousand such permuted calculations were used to plot the histogram in the inset in Figure 6. Remarkably, the *χ* ^2^ fit to the original data is smaller than all fits to the permutated datasets, indicating that the linearity assumption is meaningful and that the good fit in Figure 6 has not occurred by chance.

## Supporting Information

### Accession Numbers

The primary protein accession numbers from the Swiss-Prot databank (http://www.ebi.ac.uk/swissprot/) for the E. coli proteins mentioned in the text are: Tar MCP2 ECOLI (P07017), Tsr MCP1 ECOLI (P02942), CheW O157 CHEW ECO57 (P0A966), CheA CHEA ECOLI (P07363), and CheY O157 CHEY ECOLI (P0AE67).

## Abbreviations

E: glutamate
MeAsp: methyl-aspartate
MWC model: Monod-Wyman-Changeux model
Q: glutamine
